# The Specific Role of Relationship Life Events in the Onset of Depression during Pregnancy and the Postpartum

**DOI:** 10.1371/journal.pone.0144131

**Published:** 2015-12-08

**Authors:** Nicola Wright, Jonathan Hill, Andrew Pickles, Helen Sharp

**Affiliations:** 1 Institute of Psychology, Health and Society, University of Liverpool, Liverpool, United Kingdom; 2 School of Psychology and Clinical Language Sciences, University of Reading, Reading, United Kingdom; 3 Biostatistics Department, Institute of Psychiatry, King’s College London, London, United Kingdom; University of Rennes-1, FRANCE

## Abstract

**Background:**

The precipitating role of life events in the onset of depression is well-established. The present study sought to examine whether life events hypothesised to be personally salient would be more strongly associated with depression than other life events. In a sample of women making the first transition to parenthood, we hypothesised that negative events related to the partner relationship would be particularly salient and thus more strongly predictive of depression than other events.

**Methods:**

A community-based sample of 316 first-time mothers stratified by psychosocial risk completed interviews at 32 weeks gestation and 29 weeks postpartum to assess dated occurrence of life events and depression onsets from conception to 29 weeks postpartum. Complete data was available from 273 (86.4%). Cox proportional hazards regression was used to examine risk for onset of depression in the 6 months following a relationship event versus other events, after accounting for past history of depression and other potential confounders.

**Results:**

52 women (19.0%) experienced an onset of depression between conception and 6 months postpartum. Both relationship events (Hazard Ratio = 2.1, *p* = .001) and other life events (Hazard Ratio = 1.3, *p* = .020) were associated with increased risk for depression onset; however, relationship events showed a significantly greater risk for depression than did other life events (p = .044).

**Conclusions:**

The results are consistent with the hypothesis that personally salient events are more predictive of depression onset than other events. Further, they indicate the clinical significance of events related to the partner relationship during pregnancy and the postpartum.

## Introduction

Depression is one of the most prevalent and debilitating forms of psychopathology [1]. Impairment associated with the disorder can be severe, and the impact on the sufferer is amplified by the fact that depression is often recurrent, with each episode increasing the risk for experiencing a subsequent episode [2]. Epidemiological studies have consistently demonstrated a higher prevalence of depression in women, of one to three times higher than that of men [3].

Depression during pregnancy and the early postnatal period has been linked to a range of adverse outcomes for the developing foetus and child. Depression during pregnancy has been linked to risky health behaviours, such as substance use, tobacco and alcohol use and poor self-care [4] and health complications, such as preeclampsia [5,6] all of which can lead to negative obstetric and infant outcomes. Indeed, depression during pregnancy has been associated with poor obstetric outcomes, including low birth weight and preterm delivery [7–9]. Antenatal depression is also the strongest predictor of postnatal depression [10], which is associated with disturbances in mother-infant interactions [11, 12], later parenting disturbances [13] and problems in child cognitive, behavioural and social development [14–17].). Thus, the identification of mothers at risk for developing depression is essential to reduce the detrimental consequences of depression for the developing child. The frequent contact that pregnant women and new mothers have with health professionals provides an opportunity for identification and intervention. However, in order to identify those at risk, an understanding of the contributory factors to depression during this period is required.

The precipitating role of stress, particularly stressful life events, in the onset of major depression has received considerable research attention. Studies spanning several decades using both community and clinical populations have demonstrated that individuals with an onset of depression are significantly more likely to have experienced a recent stressful life event [18–21], with an average of 80% of episodes in women preceded by stressful life events [20]. The relationship between life events and depression has also been confirmed for depression during pregnancy (see review by Lancaster and colleagues [22]) and the postpartum (see review by Robertson and colleagues [10]). Indeed, birth itself may often act as a stressful life event [23].

An important yet largely neglected question regarding the nature of the life stress-depression relationship is whether certain types of life events are more depressogenic than others. A small body of research has examined this question, indicating that loss events [24–26] or events within the interpersonal domain [27, 28] are more strongly predictive of depression than other events. However, an important facet of life events that, despite possessing high face validity, has received little research attention is whether events that are meaningful or personally salient are more depressogenic. This premise was central to the development of the Life Events and Difficulties Schedule [29], a semi-structured interview in which an assessment of the ‘contextual threat’ of life events is conducted to produce a rating of their severity. This involves estimating the long-term implications of an event or difficulty for important plans, concerns, and purposes of the respondent. Brown and colleagues [30] tested this hypothesis using a prospective design by interviewing women to identify important areas of life commitment, for example, family or work. They found that the occurrence of a life event in the following year which ‘matched’ an area of commitment was almost three times more likely to result in onset of depression than a ‘non-matching’ event.

The method of matching events to commitments would suggest that these events were more personally salient to the women, and their findings support the hypothesis that salient events would be a stronger predictor of depression than other, less salient, events. However, another approach may be to examine a sample of individuals who are all making a similar life transition, allowing hypotheses to be made about events that may be particularly salient for that group. There has been some work with adolescents taking this approach, predicting that interpersonal events, more specifically, the end of romantic relationships, would be particularly depressogenic [31]. Consistent with predictions, the authors found romantic relationship loss events to predict depression onset in adolescents after controlling for other life events, although this was only found for first onsets and not recurrences of depression.

The transition to parenthood is a period of significant change and disruption and thus represents an emotionally turbulent time for women [32]. Mothers may need to draw on all available coping resources, especially from within the partner relationship, and thus a stable and supportive relationship would seem to be particularly important. Events which threaten or disable the partner relationship, therefore, may be particularly distressing. There is substantial evidence for an association between the quality of the partner relationship and depression in perinatal women (see reviews [10, 22]). One recent cross-sectional study examined the prediction from a number of different life events to depressive symptoms in pregnancy in a sample of 693 women. They found severe marital conflict to be one of six significant predictors of depression symptoms in their multivariate model [33].

However, most previous studies have used self-report measures of psychosocial stressors and depression symptoms in cross-section. These have several limitations including that they do not provide evidence on whether or not the stressors have preceded the depressive symptoms, nor whether the reports of the stressors reflect depressive biases. Most of the studies with perinatal samples assess depression symptoms rather than diagnosis. For example, in a recent meta-analysis of 120 studies that examined associations between modifiable partner factors (e.g. conflict and partner support) and perinatal depression, only 13 assessed depression diagnosis [34]. This is the first study to make use of an established life event methodology in which the timing of events, and of onsets and offsets of depressive episodes, are assessed by interview to examine specific psychosocial risk for depression in the transition to parenthood. Using a sample of first time mothers who were interviewed during pregnancy and at 29 weeks postpartum, we tested the hypothesis that events related to the partner relationship would show a significantly higher risk for depression onset than other types of life events.

## Method

### Ethics statement

Ethical approval for the study was granted by the Cheshire North and West Research Ethics Committee on the 27th June 2006. The letter confirming ethical agreement for the study (reference number 05/Q1506/107) stated, ‘On behalf of the Committee, I am pleased to confirm a favourable ethical approval for the above research on the basis described in the application form, protocol and supporting document as revised.’ Participants gave written informed consent.

### Sample

Participants were recruited into the Wirral Child Health and Development Study, a prospective epidemiological longitudinal study starting in pregnancy. The study used a two stage stratified design in which a consecutive general population sample of 1,233 first time pregnant women was recruited (the ‘extensive ‘ sample) and used to generate a subsample (‘intensive’ sample), for detailed study, of 316 women, stratified by psychosocial risk (see previous publication for sampling details [35]). The stratification variable, psychological abuse in current or recent relationship [36] was chosen for its known association with several risk factors for early child development. Data presented here were gathered from this intensive sample when the mothers were 32 weeks pregnant and at 29 weeks postpartum.

Socioeconomic conditions on the Wirral range between the deprived inner city and affluent suburbs, but with very low numbers from ethnic minorities. Mean age at recruitment was 27.9 years (s.d. 6.2, range 18–51) and 41.8% of the extensive sample were in the most deprived quintile of UK neighbourhoods using the English Index of Multiple Deprivation (IMD [37]).

There were 316 mothers recruited to the intensive sample at 32 weeks pregnancy, and 303 completed both the depression and life event interviews at 32 weeks, with 273 (86.4%) also completing the depression and life event interviews at 29 weeks postpartum. There were no significant differences in deprivation, risk status or maternal age between the 27 mothers who did not complete the postpartum assessment and the 273 who completed both, and they were not significantly more likely to have had a depression onset in pregnancy. The subsample of 273 was similar to the wider extensive sample in age (mean age = 27.8 years, s.d. 6.2, range 18–51) and deprivation status (37.7% in the most deprived quintile). Of the 273, 207 (75.8%) women were either married or cohabiting with a partner, 32 (11.7%) had a partner who lived elsewhere and 34 were single (12.5%). The majority, 95.2% (*N* = 260) of women described their ethnicity as White British. Within the stratified intensive sub-sample, 51% were drawn from the women with high psychosocial risk and 49% from those with low psychosocial risk.

### Procedure

Mothers completed an initial brief interview at a routine antenatal visit to the hospital responsible for their antenatal care at approximately 20 weeks gestation, which included completion of the demographic questionnaire and partner psychological abuse measure. At 32 weeks gestation mothers completed a more in depth interview to gather information on mental health, recent life events, personality functioning and relationship functioning. Depression diagnosis, assessed using the Schedule for Affective Disorders and Schizophrenia-Lifetime (SADS [38]), and life events, gathered using the Life History Calendar (LHC [38]), were used for this study. The interviews were either conducted as home visits or in the study base, by graduate Research Assistants. A similar interview was then performed at 29 weeks postpartum. The mothers were recompensed for their time with high street shopping vouchers.

### Measures

#### Demographic Questionnaire

A questionnaire was developed to collect demographic information, including marital and employment status, age and qualifications on leaving school, ethnic origin, and socio-economic status. Age and socio-economic status were included as covariates for this study. Socio-economic status was derived from post code data using the English Index of Multiple Deprivation (IMD) [37]. IMD scores were converted to quintile categories, with 1 being the ‘most deprived’ category, and 5 the ‘least deprived’.

#### Psychological Abuse Questionnaire [36]

Partner psychological abuse was assessed at 20 weeks of pregnancy using a 20-item questionnaire covering humiliating, demeaning or threatening utterances in the partner relationship over the previous year. The scale is the total from 20 no/yes (coded as 0 = absent, 1 = present) items. Participants first rated these items about their own behaviour toward their partner and then about their partner’s behaviour toward them. High and low psychological abuse strata were defined using the highest of the partner to participant and participant to partner scores for each family. A variable indicating whether the mother was high or low psychosocial risk allocation to the intensive sample was included as a covariate.

#### The Life History Calendar [39]

The LHC is a calendar-based structured interview designed to facilitate retrospective recall of life trajectories and events. The LHC comprises of a large grid, with columns representing the years and months, and rows referring to different activities (e.g. residence, employment, life events) and is marked with key events in a respondents life, such as birthdays. The interview begins by gathering information on life trajectories (e.g. where the respondent has lived) and then moves on to enquiring about specific events. This facilitates the recall of specific events by allowing the respondent to contextualise events by connecting them to other key life activities and events. The timing and duration of trajectories and events is then recorded on the calendar [39]. Reliability studies have reported agreement with previously collected concurrent data of 90% for a period of three years [39], 81% over a period of five years [40], and 69% to 79% across different trajectory categories over a period of one year [41].

In the present investigation, a list of 30 stressful life events was used with the LHC interview with a further open question allowing respondents to report any other stressful events they had experienced. Events reported under this category were included in analysis if they were present on other widely used life event checklists [42–44]. The two relationship events used were: end of romantic relationship and serious arguments with partner.

#### Schedule for Affective Disorders and Schizophrenia—Lifetime Version (SADS-L) [38]

The SADS-L is a structured diagnostic interview. The version used in this study employs an investigator-based approach and provides DSM-IV [45] based diagnoses [46]. Diagnoses of generalized anxiety disorder, panic disorder and major depression were assessed, and the major depression data used for this study. For the 32 week gestation interview, the SADS-L interview was adapted to allow ratings of DSM major depressive episodes in four lifetime periods: from one month before conception up to the 32 week interview; three years prior to conception to one month before conception; from age 16 to three years prior to conception; and before age 16. At the 29 weeks postpartum interview, ratings were made from the previous interview in pregnancy up to the current interview date. Life events and depression onsets were ascertained in the same interview and in many instances were rated by the same interviewer. However, all interviewers and coders were blind to the hypothesis that relationship life events would be more depressogenic than other types of life events.

In order to provide a constant assessment time period, depression onsets from conception to 6 months postpartum were examined for this study. A past history of depression variable was created by combining the three periods prior to pregnancy, with a positive rating in any of the periods used to indicate a past history of depression. The SADS-L was administered by trained graduate Research Assistants’, 28 audio-recordings were independently rated for reliability purposes, and weighted kappa was acceptable, ranging from .83–1.00.

### Statistical Analysis

To generate descriptive data regarding the life events preceding depression onset, the number and type of life events experienced in the 6 months prior to onset (based on [29, 47]) were examined for women who experienced an episode of depression (‘cases’). To allow comparison to the women without a depression episode (‘controls’), a random 6 month period was selected between conception and 6 months postpartum, and the number and type of life events occurring in this period was examined. The characteristics of cases and controls were estimated in SPSS.

The more formal analysis used a proportional hazards survival model [48] with time varying covariates to examine how the instantaneous risk of depression onset was associated with the relationship and non-relationship life-events in the preceding 6 months and other time fixed confounding maternal characteristics and a sample stratification factor to account for the sample design. Within the Cox proportional hazards model, the occurrence of a life event raises the risk for depression above baseline for a continuous block of 6 months after which the risk returns to baseline (unless another event occurs within that 6 months, in which case the risk is doubled for the overlapping period), therefore in the model the woman is continuously exposed to the life event for that 6 month period. Wald tests were used to assess the significance of the log hazard ratios and to test whether there was a significant difference between the risk presented by relationship events and non-relationship events. In order to display the effects graphically four conditions were illustrated, one with no life events, and three with successive 6 months periods of risk associated with life events. These models, and associated plots, were undertaken in Stata 11 [49].

## Results

Of the 273, 52 (19.0%) women experienced an onset of depression from conception to 6 months postpartum, 9 of these experienced more than one depression episode, but only first onsets were considered for this analysis. Demographic characteristics for the total sample and according to depression status are presented in Table 1. There were no significant differences between cases and controls on age at consent (*t*(273) = .66, p = .512) or deprivation χ^2^(4) = 1.41, *p* = .842). There was a significant association between past history of depression and having a depression onset in the period (χ^2^(2) = 7.49, *p* = .006) with 61.2% of cases having previously experienced an episode of depression, compared to 39.7% of controls. Further, there was a significant association between sample stratification and depression onset (χ^2^(2) = 4.68, *p* = .021), 67.3% of cases were high risk allocation, compared to 50.7% of controls.

**Table 1:** Descriptive statistics for key demographic variables and past depression history for the total sample and cases and non-cases separately.

	Cases		Controls		Total	
	Mean	SD	Mean	SD	Mean	SD
**Age**	27.27	7.10	27.83	5.94	27.72	6.17
	**N**	**%**	**N**	**%**	**N**	**%**
**Past depression**						
**Yes**	30	61.2	85	39.7	114	43.7
**No**	22	38.8	136	60.3	159	56.3
**Risk status** ^a^						
**High**	35	67.3	112	50.7	147	53.8
**Low**	17	32.7	109	49.3	126	46.2
**Deprivation** ^b^						
**1**	18	34.6	85	38.5	103	37.7
**2**	10	19.2	44	19.9	54	19.8
**3**	14	26.9	58	26.2	73	26.7
**4**	5	9.6	12	5.4	18	6.6
**5**	5	9.6	22	10	25	9.2

^a^High or low risk allocation to the sample

^b^ *=* Indices of multiple deprivation quintiles, 1 = most deprived

The descriptive statistics for both types of life event for cases and controls separately and the total sample are described in Table 2. In the total sample, the majority of women (56.7%) experienced at least one non-relationship life event in the 6 month period, with a range of 0 to 6 events reported, and a higher proportion of cases (69.2%) experiencing at least one event than controls (53.2%). In the total sample, 12.1% of women experienced at least one relationship event, with a range of 0 to 3 events reported, and more cases (26.9%) experiencing at least one relationship event than controls (8.6%). The relationship events total score was very skewed (only 2.6% endorsed more than one event) so a binary variable was used to examine the significance of the association with depression onset. A significant association was found (χ^2^(2) = 13.30, *p* = .001) with an odds ratio of 3.92. Mann-Whitney U tests were used to examine whether there was a significant difference between cases and controls in the number of non-relationship events. Cases experienced significantly more non-relationship life events (*U* (*N* = 273) = 6,941.50, Z = 2.67, *p* = .008) than controls in the 6 month period.

**Table 2:** Descriptive statistics for relationship and non-relationship life events, for the total sample and for cases and non-cases separately.

	Cases		Controls		Total	
	Mean	SD	Mean	SD	Mean	SD
**Relationship events**	0.31	0.58	0.11	0.39	0.15	0.44
**Other events**	1.58	1.65	0.93	11.2	1.05	1.26
	**N**	**%** ^a^	**N**	**%** ^a^	**N**	**%** ^a^
**Relationship events**	14	26.9	19	8.6	33	12.1
**Other events**	36	69.2	117	53.2	153	56.7

^a^Percentage of women experiencing the event at least once in the 6 month period

**Fig 1:**
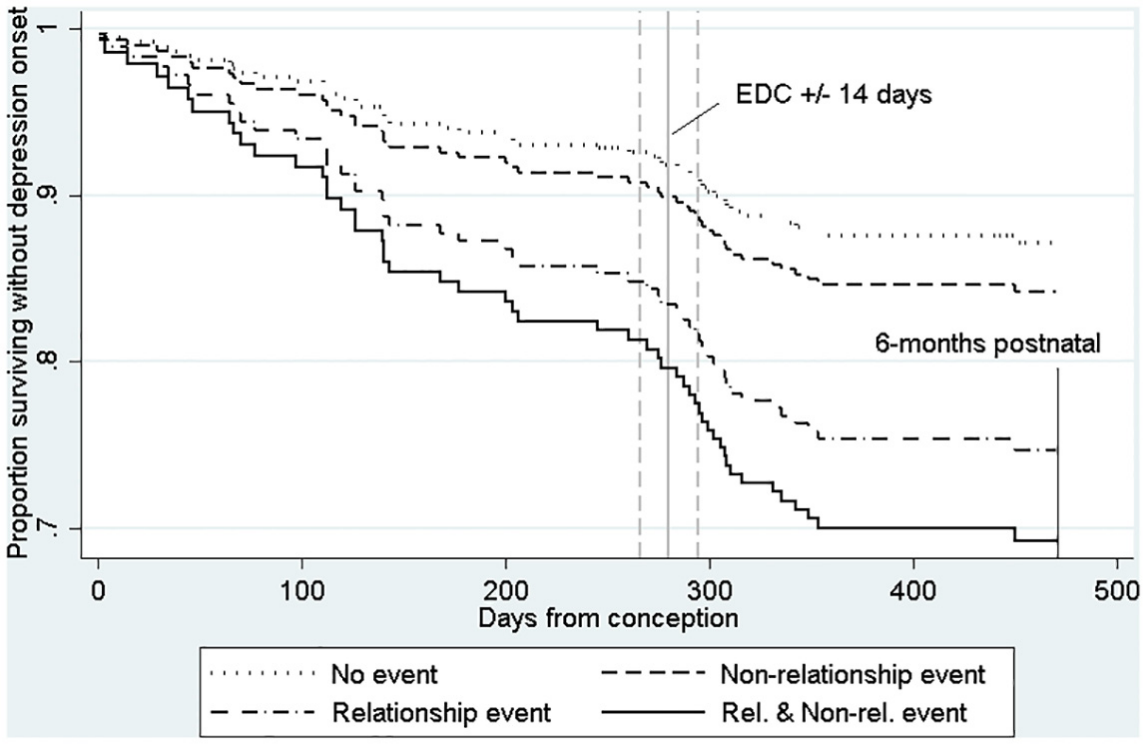
Survival plot showing model estimates for women with four different fixed life event exposures: 1) no events, 2) non-relationship event, 3) relationship event, 4) both relationship and non-relationship event.

Table 3 presents the estimated hazard ratios (instantaneous relative risks) derived from the Cox proportional hazards survival model. A history of depression prior to pregnancy substantially and significantly raised the risk of depression, but neighbourhood deprivation, maternal age and the sample stratification factor were all non-significant. Having accounted for the effects of these potential confounders, the counts over the previous six months of non-relationship and relationship events were both estimated as having large and significant effects on the risk of depression. The Wald test of the equality for the effects of the two kinds of events indicated that relationship events were significantly more depressogenic than non-relationship events (χ^2^ (1) = 4.05, *p* = 0.044). This difference is illustrated in Fig 1. This shows from the time of conception, the proportion of women remaining non-depressed for four groups generated from the proportional hazards survival model: (1) women unexposed to either kind of event, (2) women continuously exposed to one non-relationship event in the preceding 6 months, (3) as (2) but for one relationship event, and (4) women continuously exposed to one of each kind of events. The graph suggests that by delivery, the proportion having experienced an onset of depression is 9% for those exposed to a non-relationship event, but 15% for those exposed to a relationship event. By 6 months postpartum, 16% of those exposed to a non-relationship event have experienced an onset of depression, whereas 30% of those exposed to a relationship event have experienced an onset.

**Table 3:** The estimated hazard ratios (instantaneous relative risks) for relationship and non-relationship life events and the potential confounders.

	Hazard Ratio	P>z	95% CI
Non-relationship event	1.26	.020	1.04–1.53
Relationship event	2.13	.001	1.37–3.31
Past depression	2.24	.006	1.26–3.99
Deprivation	1.14	.225	0.92–1.42
Maternal age	.99	.643	0.94–1.04
Risk stratum	1.28	.613	0.49–3.31

## Discussion

This study examined whether events hypothesised to be particularly salient for women making the first transition to parenthood would be a stronger predictor of depression onset than other types of life events. The results supported this hypothesis; whilst both types of events were significantly associated with risk for depression onset, the occurrence of a relationship event in the preceding six months was a significantly stronger predictor of depression onset than was the occurrence of other types of life events. This was found after accounting for past history of depression and other potential confounding variables.

Both the non-relationship and relationship life events were associated with risk for depression, consistent with the substantial literature documenting an association between life events and depression onset [18–21]. However, the current study was novel in demonstrating that events related to the partner relationship were associated with a significantly higher risk for depression onset than were other types of events in a sample of perinatal women. The stronger prediction from relationship events is consistent with the literature which has documented associations between relationship quality and depression in perinatal women (see reviews [10,22]).

The findings are consistent with the hypothesis that life events which are more personally salient will be stronger predictors of depression onset than other life events. Previously, Brown and colleagues [30] demonstrated that the occurrence of life events which ‘matched’ an important area of life commitment were three times more likely to precipitate onset of depression than were other events. Further, Monroe and colleagues [31] hypothesised that the end of romantic relationship would be particularly meaningful for adolescents, and found that after controlling for 14 other life events from the Schedule of Recent Experiences [50] and daily hassles assessed using the Unpleasant Events Schedule [51], end of romantic relationship significantly increased the prediction of first but not recurrent onsets of depression In the present study it was theorised that for women making the first transition to parenthood, events within the partner relationship would be particularly salient. The first transition to parenthood is a period of great emotional upheaval and so support and stability in the partner relationship would seem to be of particular importance. The results were consistent with the prediction that events which threaten the stability or mark the end of the partner relationship would be particularly depressogenic for a group of perinatal women.

The clinical significance of relationship events in perinatal depression is consistent with the theoretical underpinnings of Interpersonal Psychotherapy (IPT) for depression. IPT is rooted in attachment and interpersonal theory and is based on the premise that experiencing social disruptions increases the risk for depression [52]. A number of randomized controlled trials have provided support for the use of IPT for perinatal depression [53–55].

A strength of the current study lies in its prospective design, with two interview periods used to recall life events over a maximum period of 8 months, using the LHC, which has been demonstrated to yield reliable recall for longer periods of time [39]. This afforded some prospective prediction, with life events collected at the initial interview being used to predict onsets of depression reported at the second interview. The use of the LHC interview, which dates the occurrence of life events, also allowed that only events which were reported to have occurred before depression onset were considered for analysis. Failure to confirm this has been a problem in life event research using checklists [3] particularly in research with perinatal samples [56]. Ensuring that events precede depression onset is critical for examining the aetiological importance of relationship events, as evidence suggests that depression generates the occurrence of such events [57].

The reported findings should also be viewed in light of several limitations. Firstly, the life event and depression interviews were conducted in the same interview session, which allows the possibility of reporting bias leading to inflated associations. However, as this should affect relationship and non-relationship events equally it would not explain the different associations found for the two types of events. Secondly, as both the life event and depression interviews were administered by the same interviewer, and typically the same interviewer would administer the pregnancy and postpartum interview for each mother, the depression ratings were not always made independently of life event information, although it was ensured that all interviewers and raters were blind to the study hypothesis. Thirdly, a measure of relationship abuse administered during pregnancy was used to stratify the sample; this benefited the analysis by ensuring a sufficient number of relationship events within the time period examined. However, it does limit the generalisability of the findings, as 51% of the intensive sample were high on psychosocial risk. Stratification status was controlled for in all analyses but replication of the findings in a true general population sample would be desirable, particularly given the low representation of ethnic minority groups in the current sample. Finally, the study design does not allow us to evaluate the contribution of relationship life events in relation to other likely causes, notable genetic factors, nor does it account for heterogeneity within depressive disorders.

The identification of a specific type of life event as particularly depressogenic for women making the first transition to parenthood has important implications for practice. Pregnant and postpartum women have increased contact with health professionals, but may be less likely to seek help for depression due to the social stigma attached to depression experienced during this period [58]. The identification of specific risk factors for depression onset facilitates identification of those at risk and allows early intervention. Recent international clinical guidelines outlined the importance of conducting psychosocial risk assessments with perinatal women [59] and the current findings further underscore the importance of screening for the occurrence relationship events. As previously noted, the findings may also lend support for the use of IPT in the treatment of perinatal depression.
